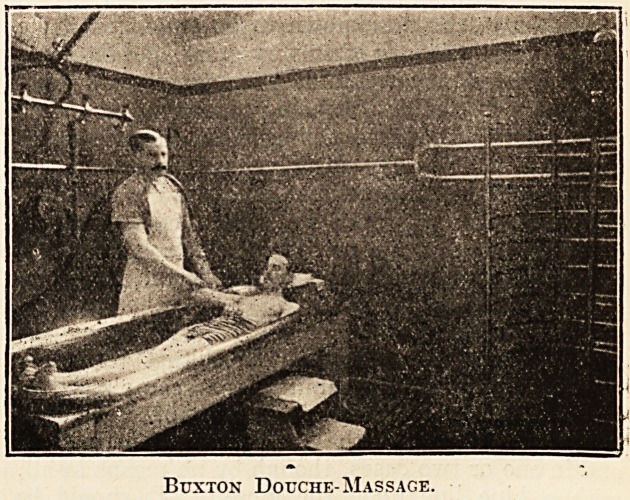# Buxton

**Published:** 1911-03-25

**Authors:** 


### HOME AND CONTINENTAL SPAS.

### III.—BUXTON.

Situated in the centre of the Peak District of Derbyshire, Buxton is popularly known as "The Mountain Spa." The town, which is the highest in the United Kingdom, being a thousand feet above sea level, is singularly well-kept and possesses many stately buildings and pleasant gardens. Owing to the limestone formation its roads and streets dry very rapidly, even after the heaviest showers, and this probably is one of the reasons for the exceptional cleanliness which Buxton presents to the eye of the visitor. The Spa is 163 miles from London by the most direct route, which is that of the Midland Railway, and is reached in four hours from St. Pancras, the London terminus of the Company. It covers an area of 1,275 acres and has an ordinary population of 12,200 inhabitants. The surrounding country is typical of the Peak District, being exceptionally grand and bold of outline. In clear weather many extensive and delightful views are to be obtained from the breezy uplands; but the most picturesque and enchanting of its scenery is in the dales.

General View of Buxton.

### Climate and Season.

The climate is remarkably dry and bracing, and, in consequence of the altitude, the air is peculiarly rarified, light, and exhilarating. The rainfall is above the average, being 46.2 inches, but owing to the hilly character of the town the surface drainage is very rapid, and the limestone rocks being non-absorbent, all the drawbacks of a damp subsoil are practically non-existent. The record of sunshine is usually very high; in 1909 it was over 1,519 hours. The temperature is always a few degrees cooler than the lowlands, a feature which is not unpleasant in summer, whilst in the winter the dryness of the air renders the keenest frost bracing and enjoyable. May to October comprises the Buxton season, and at this time the gardens and terraces are gay with flowers. The covered sidewalks and promenades are hung with ferns and baskets of flowers, which do much to make the Spa bright and attractive. Although the season is essentially a summer one, the winter months, owing to the usually heavy snowfall, afford such excellent, and in this country exceptional, sports as sleighing, ski-ing, tobogganing, skating, and curling. A new Swiss toboggan run is now being erected, which, when complete, will be the only one of its kind in the British Isles.

### History.

It is evident, from the remains of the Roman villas, baths, and other buildings that have been found from time to time in the district that the healing virtues of Buxton's thermal springs were known to, and appreciated by the Romans. The neolithic barrows in the neighbourhood have yielded interesting relics of even greater age, and it is claimed that the reputation of the waters descends through the centuries unimpaired from prehistoric times. In the early part of the Christian era the springs were dedicated to St. Anne, who "gave health and living great to those who love her most." The town is mentioned in the Domesday Survey as Bawkestones, but it is not until the Tudor period that Buxton really begins to appear in important records. Soon after the accession of Queen Elizabeth the baths and wells became very popular and were resorted to by the nobility and gentry. It was at this time that Dr. John Jones, an eminent physician of the period, took charge of the Spa, and published, in 1572, the first handbook on its waters, under the title of "The Benefite of the Ancient Bathes of Buckstones." His most illustrious patient was Mary Queen of

Scots, who visited Buxton at least four times, and on the last occasion, in 1583, she is said to have inscribed upon one of the windows of the room she occupied the following couplet:—

> " Buxtona quæ calidæ celebrabere nomine lymphæ

> Forté mihi posthac non adeunda, vale ! "

which to-day is included in the arms of the town and may be thus translated:—

> " Buxton, whose fame thy milk-warm waters tell,
> Whom I, perhaps, no more shall see, farewell ! "

Unfortunately, all documentary evidence, registers of Dr. Jones, were destroyed 100 years later, and, in consequence we are doubtless deprived of much

The Pump Room : Exterior.

interesting and curious information concerning Buxton and its waters. In 1784, a century afterwards, the Crescent and other important buildings, which remain to the present day, were erected by the fifth Duke of Devonshire, in order to meet the demands of the growing popularity of the Spa.

The Springs, their Nature and Constituents.

The thermal springs, which are the property of the town, issue in the valley near the bathing establishments. The waters are of the uniform temperature of 82° F., beautifully bright, soft, and clear, and, when seen in the mass, of a light-blue colour. A recent analysis gave the following constituents:—

| | Grains per Gall. |
|---|---|
| Bicarbonate of Calcium ... ... ... | 14·10 |
| ,, Magnesium ... ... | 6·02 |
| ,, Iron ... ... ... | ·03 |
| ,, Manganese ... ... | ·03 |
| Sulphate of Barium ... ... ... | ·05 |
| ,, Calcium ... ... ... | ·26 |
| ,, Potassium ... ... ... | ·62 |
| ,, Sodium ... ... ... | ·84 |
| Nitrate of Sodium ... ... ... | ·03 |
| Chloride of Calcium ... ... ... | ·02 |
| ,, Sodium ... ... ... | 3·10 |
| ,, Ammonium ... ... ... | trace |
| ,, Magnesium ... ... ... | ·95 |
| Silicic Acid ... ... ... ... ... | ·95 |
| Organic Matter ... ... ... ... | ·02 |
| Carbonic Dioxide ... ... ... ... | ·20 |
| Nitrogen ... ... ... ... ... | ·19 |
| Lithium, Strontium, Lead, Phosphoric Acid | traces |
| | 27·32 |

| | Per gallon, cubic inches | Per centage Composition |
|---|---|---|
| Nitrogen ... ... ... | 6·1 ... | 59·78 |
| Carbonic Acid Gas... ... | 4·1 ... | 40·22 |
| | 10·2 | 100·00 |

The mud deposited around the mouth of the springs showed traces of cobalt and molybdenum; the latter element has never been found previously in any mineral spring. The free gases, nitrogen and carbonic acid, in the nascent state, and the recent demonstration by Lord Rayleigh of the presence of argon and helium, and by Lord Blythswood of radium, may, to some extent, explain the therapeutic action of the waters. The chalybeate spring is used both for bathing and drinking purposes, especially in cases of anæmia, a condition which is usually greatly benefited by a course of treatment at Buxton. Recent analysis of the chalybeate spring gave the following results:—

| | Grains per ga |
|---|---|
| Proto-carbonate of Iron ... ... ... | 3·[illegible]6 |
| Alumina ... ... ... ... ... | 1·18 |
| Sulphate of Manganese ... ... ... | traces |
| Silicia ... ... ... ... ... | 1·22 |
| Sulphate of Calcium ... ... ... | 9·11 |
| ,, Magnesia ... ... ... | 4·90 |
| Carbonate of Magnesia ... ... ... | 1·98 |
| Sulphate of Potash ... ... ... | traces |
| Chloride of Potassium ... ... ... | 1·40 |
| ,, Sodium ... ... ... | 2·10 |
| Sulphate of Sodium ... ... ... | 1·89 |
| Organic Matter, etc· ... ... ... | 0·36 |
| | 27·50 |

It will be seen that the iron is present as a protocarbonate, a form in which it is usually much more easily borne by the stomach. Both the thermal and chalybeate waters are dispensed for drinking in the pump room, which was erected in 1894 over St. Anne's Well. Here the " cure " may be taken

The Pump Room : Interior.

daily, including Sundays, at any time during which the well is open.

The Baths.

The bathing establishments, which are controlled by the Town Council, occupy a central position within a mile of any part of the town. They are in two sections, the natural and the hot baths, both of which are supplied with water from the thermal springs. In the natural baths, which consist of douche-immersion and swimming baths for both sexes, the waters are administered at the natural temperature of 82° F. The swimming-baths strikingly exhibit the beautiful blue colour of the water, and bubbles of the gases with which it is charged may be seen rising to the surface. These baths are paved with slabs of perforated marble, through which a stream of water constantly flows direct from the springs.

This section also includes an inhalation room, fitted with special appliances for the employment of the mineral waters in the treatment of diseases of the throat, ear, nose, and eyes. The hot baths, where the waters are raised to any temperature that may be prescribed, comprise every form of modern hydro-therapeutic apparatus, and include Aix, Vichy, and

Ladies' Swimming-Bath.

Plombières douches, Bourbon, Nauheim, Fango Mud, vapour, Dowsing, light, and electric treatments. A special feature of this establishment is the Buxton Douche-Massage bath, which has been in use for many years, and the increasing demand for it has necessitated the installation of additional baths. Considerable structural alterations in the establishments, and many very important improvements in the mechanical appliances, have been effected under the present manager, Mr. Whitworth Crone, and, in consequence of the inquiries for mud and chalybeate baths, a department has recently been added where it is possible to secure all the advantage of the Continental "Moorbader," in the form most approved of at Marienbad, Homburg, and Carlsbad.

### Diseases Treated.

The climate and waters of the Spa are of marked assistance in the treatment of gout, rheumatism, rheumatoid arthritis, sciatica, muco-membranous colitis, nervous disorders, neurasthenia, phlebitis, arterio-sclerosis, anæmia, disorder of the digestion, and skin diseases, especially those of gouty origin. Buxton has also a considerable reputation for the after-treatment of malaria and other tropical diseases. The bracing climate and the use of the chalybeate waters for drinking and bathing purposes are especially beneficial in anæmia and convalescence after prolonged illness. The baths are open and the waters are equally efficacious all the year round; the authorities, however, warn visitors against the use of the mineral waters without proper medical advice, a rule which is worthy of imitation by other Spas of this country. The cost and duration of the "cure" depend very largely upon the nature of the disease treated and the class of baths required. A course of ordinary immersion baths can be had for the modest sum of 6s. 6d. per week, and as a general rule the duration of a bathing "cure" is from twenty-one to forty-two days.

### Diet.

The Buxton Medical Society has drawn up special diet charts for the guidance of patients undergoing the treatment, which may be modified to suit the requirement of individual cases. The most specialised forms of dietary are also obtainable in most of the hydropathic establishments and other institutions for which the Spa is especially noted.

### Accommodation.

Of first-class hotels and hydros there is a fair number, and the arrangements for the comfort of invalids are in the highest degree satisfactory.* For the more modest purse there is an abundance of comfortable boarding and apartment houses within a few minutes' walk of the baths, where excellent accommodation can be obtained at the inclusive cost of 40s. to 50s. per week. For infirm patients and those who have not the free use of their limbs, hackney carriages, taxicabs, and bath chairs may be hired from ranks in the principal thoroughfares, but the doctors of the Spa always encourage patients to walk whenever it is possible so to do.

### Relaxations and Amusements.

The Spa provides exceptional opportunities for entertainment and recreation, no matter what the weather may be like. In the Pavilion Gardens, which cover an area of 23 acres, practically adjoining the bathing establishments, are ornamental and boating lakes, a terrace promenade, pretty cascades

Buxton Douche-Massage.

formed by the Wye, bowling greens, one of which is said to have been in existence in the days of Queen Elizabeth, tennis and croquet lawns, and an open-air skating rink. Here visitors congregate in the early morning after taking the "cure" to enjoy the delightful music rendered by an excellent orchestra.

* For particulars of hydros and hotels see page xxxi.

In the evening the gardens are illuminated with many-coloured electric lights, producing a most charming effect of transporting the visitor to a veritable fairyland. The Pavilion is a miniature "Crystal Palace," containing fine conservatories ornamented with costly palms and flowering plants, and a spacious concert hall capable of accommodating over 2,000 people. In this hall a band plays in the morning and evening if the weather is unfavourable for outdoor promenades, and concerts are given from time to time, for which the leading popular artists are engaged. There is also a handsome Opera House and theatre adorned with delicately-painted ceilings and panels, and lavish with marble in the staircases and box pillars, and an important feature is the fact that an uninterrupted view of the stage can be obtained from every seat in the house. First-class theatrical and opera companies are booked here throughout the year. In the park, the grounds of which cover an area of 120 acres, are afforded opportunities for cricket in the summer and lacrosse in the winter months. There is an eighteen-hole golf course on Fairfield Common, within fifteen minutes' walk, and a nine-hole golf course within five minutes' walk, of the centre of the town. The moors in the neighbourhood provide grouse shooting, and fishing may be enjoyed in the river Wye, which yields good trout. Enjoyable coaching trips are run in the summer months to many famous places within easy reach of Buxton, such as Haddon Hall and Chatsworth House, the Derbyshire seat of the Dukes of Devonshire.

### The Devonshire Hospital.

No description of Buxton can be complete without mention of the Devonshire Hospital and Buxton Bath Charity, which occupies an extensive range of buildings, granted by the sixth Duke of Devonshire, at the nominal rental of 5s. per annum, for the use of the sick poor for ever. The Hospital is no local institution, but a national hospital instituted for the relief of the poor from all parts of the United Kingdom, suffering from rheumatism, gout, sciatica, rheumatoid arthritis, and allied diseases. There are suites of baths erected near the hospital for the exclusive use of the patients, and no fewer than 34,336 mineral-water baths were given and 3,378 patients admitted for treatment during the past year, of whom 3,024 were discharged as "improved." These results afford the strongest testimony of the curative properties possessed by the waters of the Spa.

An interesting and striking feature of the hospital is the central hall, which is capable of seating several thousand people, and provides the patients with a covered, dry, warmed area of half-an-acre, canopied by the largest dome in the world, where exercise and recreation may be obtained in doubtful weather.

## Figures and Tables

**Figure f1:**
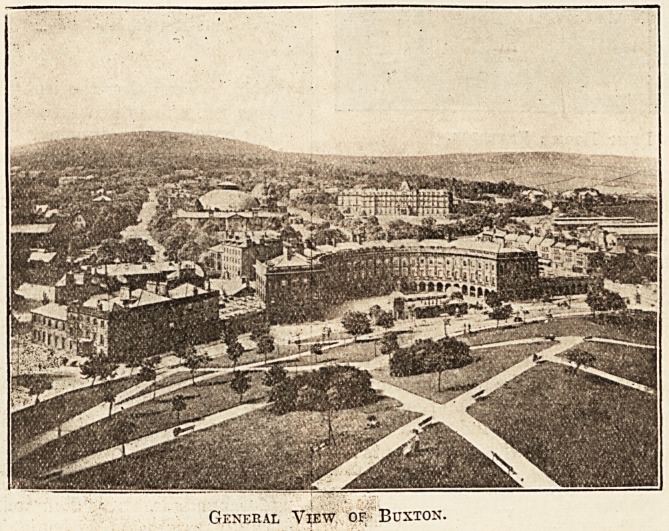


**Figure f2:**
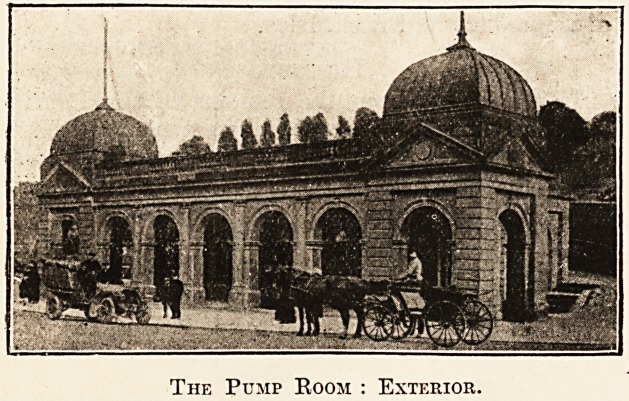


**Figure f3:**
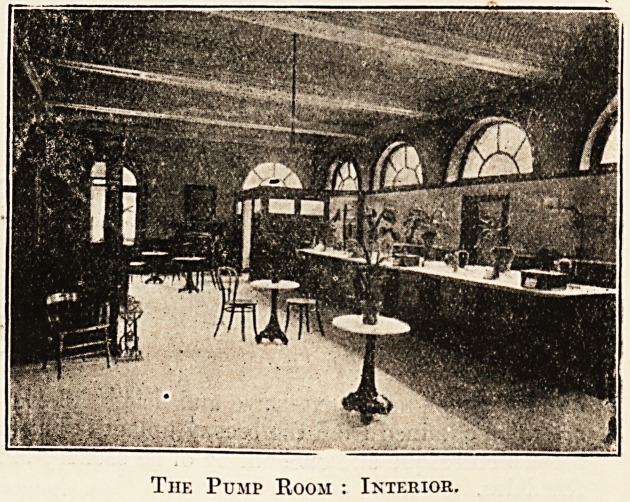


**Figure f4:**
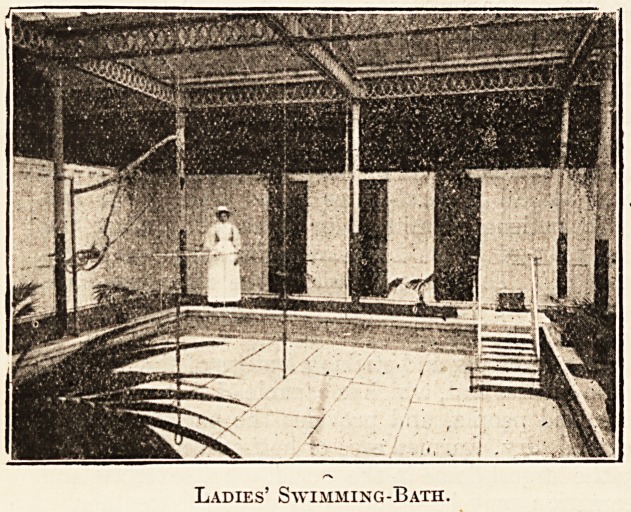


**Figure f5:**